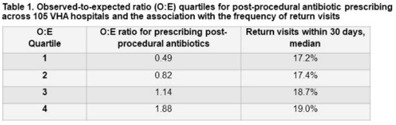# Antibiotic-prescribing practices and associated outcomes after common urologic procedures in an integrated healthcare system

**DOI:** 10.1017/ash.2022.94

**Published:** 2022-05-16

**Authors:** Daniel Livorsi, Bibiana Ruiz Granado, Bruce Alexander, Ryan Steinberg, Vignesh Packiam, Brian Lund

## Abstract

**Background:** Many urologists continue antibiotics after common urologic procedure beyond the timeframes recommended by professional guidelines. In this study, we sought to evaluate the association between postprocedural antibiotic use and patient outcomes. **Methods:** We identified all patients who underwent 1 of 3 urologic procedures (transurethral resection of bladder tumor [TURBT], transurethral resection of prostate [TURP], and ureteroscopy) within the Veterans’ Health Administration (VHA) between January 1, 2017, and June 30, 2021. A postprocedural antibiotic was any antibiotic potentially used for a urinary tract–related indication that was prescribed for administration after the day of the procedure. Outcomes were captured within 30 days of the procedure and included (1) return visits, defined as any emergency department or urgent care encounter or hospital readmission, and (2) *Clostridium difficile* infection (CDI), defined as a positive test for *C. difficile* and the prescription of an anti-CDI antibiotic. We used log-binomial models with risk adjustment to determine the association between postprocedural antibiotic use and outcomes. We constructed hospital-level observed-to-expected ratios for postprocedural antibiotic use, and we used these models to calculate the probability of each patient receiving postprocedural antibiotics. **Results:** Overall, we identified 74,629 patients; 98% were male; the mean age was 70 years (SD, 10). Among them, 50% underwent TURBT, 28% underwent TURP, and 23% underwent ureteroscopy. A postprocedural antibiotic was prescribed to 25,738 (35%) cases for a median duration of 3 days (IQR, 3–6). Return visits occurred in 13,489 patients (18%), and CDI occurred in 104 patients (0.1%). Patients exposed to postprocedural antibiotics had 16% more return visits (RR, 1.16; 95% CI, 1.13–1.20) and more than twice as much CDI (RR, 2.22; 95% CI, 1.51–3.26) than patients not exposed to postprocedural antibiotics. In log-binomial risk-adjusted analysis, the risk of return visits did not differ between the 2 groups (RR, 1.00; 95% CI, 0.97–1.04) but the risk of CDI was higher in patients who received post-procedural antibiotics (RR, 1.87; 95% CI, 1.00–3.51). Hospitals (n = 105) varied widely in their observed-to-expected ratios for prescribing postprocedural antibiotics, and the frequency of return visits was similar regardless of the frequency at which postprocedural antibiotics were prescribed (Table 1). **Conclusions:** Postprocedural antibiotics were prescribed beyond recommended intervals after more than one-third of common urologic procedures, with a large degree of variability across hospitals. The use of postprocedural antibiotics was not associated with fewer return visits but was associated with a nonsignificant increase in CDI risk. Efforts to reduce postprocedural antibiotics are needed.

**Funding:** Yes

**Disclosures:** This work was funded, in part, by the Merck Investigator Studies Program. This work was also supported by a Career Development Award (DJL) from the VA Health Services Research and Development Service (CDA 16-204) and by the Iowa City VA Health Care System, Department of Pharmacy Services.

**Table:**